# Biological denitrification of brine: the effect of compatible solutes on enzyme activities and fatty acid degradation

**DOI:** 10.1007/s10532-012-9542-0

**Published:** 2012-01-29

**Authors:** Paweł Cyplik, Agnieszka Piotrowska-Cyplik, Roman Marecik, Jakub Czarny, Agnieszka Drożdżyńska, Łukasz Chrzanowski

**Affiliations:** 1 Department of Biotechnology and Food Microbiology, Poznań University of Life Sciences, Wojska Polskiego 48, 60-627 Poznań, Poland; 2 Institute of Food Technology of Plant Origin, Poznań University of Life Sciences, Wojska Polskiego 31, 60-624 Poznań, Poland; 3 Institute of Forensic Genetics, Al. Mickiewicza 3/4, 85-071 Bydgoszcz, Poland; 4 Institute of Chemical Technology and Engineering, Poznań University of Technology, Pl. M. Skłodowskiej-Curie 2, 60-965 Poznań, Poland

**Keywords:** Compatible solutes, Denitrification, Fatty acids, Lipase, Nitrate reductase

## Abstract

The effect of the addition of compatible solutes (ectoine and trehalose) on the denitrification process of saline wastewater was studied. In saline wastewater, it was observed that the initial concentration of nitrates was 500 mg N l^−1^. A fatty substance isolated from oiled bleaching earth (waste of vegetable oil refining process) was used as a source of carbon. The consortium, which was responsible for the denitrification process originated from the wastewater of the vegetable oil industry. The consortium of microorganisms was identified by the use of restriction fragment length polymorphism of 16S rRNA gene amplicons and sequencing techniques. It was noted that ectoine affects significantly the activity of lipase and nitrate reductase, and resulted in faster denitrification compared to saline wastewater with the addition of trehalose or control saline wastewater (without compatible solutes). It was observed that relative enzyme activities of lipase and nitrate reductase increased by 32 and 35%, respectively, in the presence of 1 mM ectoine. This resulted in an increase in specific nitrate reduction rate in the presence of 1 mM ectoine to 5.7 mg N g^−1^ VSS h^−1^, which was higher than in the absence of ectoine (3.2 mg N g^−1^ VSS h^−1^). The addition of trehalose did not have an effect on nitrate removals. Moreover, it was found that trehalose was used up completely by bacteria as a source of carbon in the denitrification process. The fatty acids were biodegraded by 74% in the presence of 1 mM ectoine.

## Introduction

A common problem with wastewaters is that one of the components is usually predominant, which makes their utilization challenging. This can be illustrated by the example of saline wastewaters with a high content of nitrates originating from the production of explosive materials (Glass and Silverstein 1999), chemical fertilizers (Leakowić et al. 2000), regeneration of ion-exchange resins (Cyplik et al. 2010) and others. They can be characterized by a marginal content of phosphorus, microelements, and most importantly, a source of organic carbon, which is concurrently a source of energy for the denitrification (Cyplik et al. 2012). Moreover the high osmotic potential of brines usually limits the possibility of introducing microorganisms for biological treatment. The presence of sodium chloride and oxyanions at concentration above 2,000 mg/l create additional problems that hamper this process.

Therefore the denitrification process of brines requires the external supplementation with carbon source, which can be simply realized by combining wastes from vegetable oil production.

The fat industry represents one of the branches of the food industry that produces waste materials that are very difficult to biodegrade (Arvanitoyannis and Kassaveti 2007; Chandra and Sathiavelu 2009). Fatty waste products are difficult to utilize because of the large amounts of contaminants they contain that can subsequently contaminate water and soil. Among the various types of waste products from the vegetable oil industry, oiled bleaching earth creates the biggest problem for their utilization. It contains from 10 to 60% fatty substances, depending on the applied technology. In Poland, fat industry plants produce annually 40 kt of oiled bleaching earth, and it is assumed that this amount may increase. Previous studies on the biodegradation of biodiesel under anaerobic conditions (Cyplik et al. 2011) confirmed that fatty acid methyl and ethyl esters may be used as a carbon source, which does not alter the community structure and biodegradation potential. The application of fatty substances to enhance the denitrification efficiency of high salinity wastewater with a high content of nitrates seems like a plausible strategy to resolve the above-mentioned issues.

An important physiological mechanism of microorganisms that determines whether they can function in a particular habitat, is their ability to react to osmotic changes in the environment. The growth of microorganisms in an environment with low water activity is possible in part because of the cellular synthesis or intake of saccharides, alcohols and amino acids from the environment, which stabilize and protect cells from high salinity (Pastor et al. 2010). Compatible solutes contain a suitable composition of cytoplasm, and they increase the cell’s osmotic potential, and result in the maintenance of an appropriate turgor pressure. Compatible solutes stabilize the structure of proteins and nucleic acids (Argüelles 2000; Pastor et al. 2010). In addition, they also level off the differences between the osmotic pressure of the cell and the osmotic pressure of the environment (Roessler and Müller 2001).

Compatible solutes are amphiphilic molecules capable of increasing the total water content, as well as the cytoplasmic volume of cells, what might be responsible for reverse osmotic inhibition (Cayley et al. 1992). As proposed by Wiggins 1990, the structure-forming and breaking properties of compatible solutes may indirectly influence the hydration shells and thus the activities of the proteins involved.

Ectoine is a substance which is considered as a major protein stabilizer. Trehalose regulates the osmotic pressure both in the environment and inside the cell. It is accumulated in the periplasm and cytoplasm. The presence of trehalose in the cellular membranes decreases the temperature of phase transitions for lipids. This enables the liquid–crystal membrane structure to be maintained despite water deficiency or a notable drop of temperature (Leslie et al. 1995). Unlike ectoine, the synthesis of trehalose is dependant on the presence of nitrogen and compatible solutes in the environment.

Because the denitrification process was carried out in an environment with increased osmotic potential, the ability of bacteria to synthesize and accumulate compatible solutes was studied. The effect of compatible solutes on the denitrification process and the biodegradation of fatty acids was determined as well.

## Experimental procedures

### Composition of wastewater

The composition of synthetic wastewaters is presented in Table 1. In wastewaters, the initial concentration of nitrates was 500 mg N l^−1^ (pH 7.2). Fatty substances (1.5 g l^−1^) were used as a carbon source (C:N = 3).

**Table 1:** Composition of synthetic wastewater

Parameter	g l^−1^
NO_3_^—^N	0.5
Compatible solutes (1 mM):	
Ectoine	0.142
Trehalose	0.342
KH_2_PO_4_	2.8
KH_2_PO_4_	7
NaCl	15
MgSO_4_·7H_2_O	0.01
FeSO_4_·7H_2_O	0.001
MnSO_4_·4H_2_O	0.0005
ZnCl_2_	0.00064
CaCl_2__·_6H_2_O	0.0001
BaCl_2_	0.00006
CoSO_4_·7H_2_O	0.000036
CuSO_4_·5H_2_O	0.000036
H_3_BO_3_	0.00065
Na_2_MoO_4_	0.005
EDTA	0.001
C12	0.036
C14	0.028
C16	0.66
C18	0.070
C18:1	0.625
C18:2	0.075

### Microorganisms and their genetic identification

A consortium of microorganisms isolated from a biological waste treatment plant was used in these studies. Identification of species included in this mixed population of microorganisms was achieved by the PCR–RFLP technique and sequencing. Total bacterial DNA was isolated using the Genomic Mini kit from A&A Biotechnology (Gdynia, Poland). A mixture of 16S rDNA was amplified on a matrix of genomic DNA. The PCR reaction was carried out in volume of 25 μl in a Biometra T Gradient apparatus. The reaction mixture contained 100 ng genomic DNA, 1 × reaction buffer (10 mM Tris–HCl, 50 mM KCl, 0.1% Triton X-100, 2.5 mM MgCl_2_), 200 μM dNTP, 0.44 μM of each primer oligonucleotide, 2 U DNA polymerase (DyNAzyme II, FinnZymes). The thermal cycling profile was: initial denaturation at 95°C for 5 min, hybridization at 48°C for 1 min, elongation at 72°C for 1 min, 30 cycles. The following oligonucleotide primers were used in the reaction: SD-B 5′AGAGTTTGATCCTGGCTCAGT3′ and SD-U 5′ACGGCTACCTTGTTACGACTT3′.

PCR amplicons were purified with the Clean-up kit (A&A Biotechnology), and cloned into the pGEM-T Easy vector (Promega, Madison, USA). Chemically competent cells of *E. coli* JM109 (Promega) were transformed with this vector. Transformants were cultured for 16 h at 37°C in LB (Luria–Bertani) medium with agar (Difco, USA) and ampicillin at a concentration of 100 μg ml^−1^. One hundred microliters of 100 mM isopropyl-β-d-thiogalactoside and 20 μl of 50 mg ml^−1^ bromo-chloro-indolyl-galactopyranoside (X-gal) were plated on Petri dishes. Plasmids were isolated with the use of the Plasmid Mini Kit (A&A Biotechnology). Reamplification of 16S rDNA was carried out on a matrix of plasmid DNA. PCR amplicons were digested with the restriction enzymes, HinfI and TaqI. Products of the digestion were separated by electrophoresis in a 2% agarose gel in 1× TBE buffer. DNA in the gel was visualized by the addition of ethidium bromide at 5 μg ml^−1^. Plasmid DNA that was not subjected to restriction digestion was sequenced (Genomed Warszawa, Poland). The results from sequencing were analyzed with the use of VectorNTI software, and BLASTN and Ribosomal Database Project databases.

### Culture conditions

Loosely capped 250-ml Duran-Schott bottles contained 50 ml of wastewater. The initial inoculum was adjusted to an optical density of 0.10 ± 0.03 at 600 nm, and the bacteria were cultured at 25°C and 100 rpm. Limited aeration was achieved by flushing with nitrogen to remove dissolved oxygen from the medium and the headspace (Cyplik et al. 2011). All the experiments were carried out in three replicates.

### Determination of nitrate reductase activity

The activity of nitrate reductase was determined based on measurements of the amount of nitrites produced from nitrates during the enzymatic reaction. Reaction was carried out at pH 7.2. Samples were incubated at 37°C for 15 min. A sample of 100 ml was taken from the culture and centrifuged at 4,000×*g*. Obtained biomass was suspended at 0.1 M phosphate buffer (pH 7.2) containing 1% NaCl, then centrifuged. This step was repeated twice. The biomass was condensed to the final volume 20 ml. Then, the cells were broken with the use of glass beads of 0.3 mm diameter for 30 min in a bead mill. After the cells were broken and separated from the glass beads, the entire sample was centrifuged at 5,000×*g*. A reaction mixture with following composition was prepared in a vial to a final volume of 3 ml: 1 ml KNO_3_ (100 mM), 0.5 ml Tris-HCI (100 mM), 0.5 ml methyl viologen (dichloride 1,1′–dimethyl-4,4′–bipyridine) (120 μM), 1 ml NaCl (1 M) and 50 μl crude extract. The reaction was initiated by the addition 0.1 ml sodium hydrogen sulfite (0.5 M). Samples were incubated for 15 min in a water bath at 37°C. Then, 0.1 ml formaldehyde was added to stop the reaction.

### Determination of lipolytic activity

Lipolytic activity was determined based on the hydrolysis reaction of p-nitrophenyl phosphate (p-NPP) (Lotrakul and Dharmsthiti 1997). Enzymatic activity was calculated as the amount of μM p-NP released within 1 h, at 37°C from 1 ml of culture. The reaction mixture was composed of 540 μl of solution A (2 mM p-NPP dissolved in 2-propanol) and 4,860 μl of solution B (4 g Triton × 100 and 0.1 g Arabic gum in 100 ml phosphate buffer, pH 7.2). The mixture was emulsified for 10 min in an ultrasonic bath, and then heated for 5 min at 37°C in a water bath. After an initial warming of the mixture, 1 ml of culture was added. The entire mixture was incubated for 15 min at 37°C. The sample was centrifuged at 3,600×*g* for 15 min at 25°C.

### Determination of nitrates and nitrites

The nitrate content of the brine was determined by spectrophotometry via reaction with sodium salicylate. The amount of produced nitrites in a reaction mixture, equal to the amount of reduced nitrates, was determined spectrophotometrically (λ = 530 nm) with sulfanilic acid and 1-naphthylamine. Volatile suspended solid (VSS) was determined using a standard method (Hermanowicz 1976).

### Determination of trehalose, glycerol and ectoine

Compatible solutes from cells were isolated according to the methods described by Onraedt et al. (2004); Roustan and Sablayrolles (2002). Trehalose and glycerol were determined by HPLC (1200 series, Agilent Technologies, Santa Clara, CA). Analysis was performed isocratically at a flow rate 0.6 ml min^−1^, at 60°C on an Aminex HPX-87H 300 × 7.8 mm column (Bio-Rad, Hercules, CA). The mobile phase consisted of 0.001 N H_2_SO_4_. Thirty microliter samples were injected into the column.

Determinations of ectoine were carried out on the same HPLC apparatus. Analysis was performed isocratically at a flow rate of 2 ml min^−1^, at 70°C, on a Nucleosil 100-5 NH_2_ column (Mecherery-Nagel, Germany). The mobile phase was acetonitrile:water (70:30). Samples were filtered through 0.22 μm Millex-GS filters (Millipore, USA), and were injected in a volume of 30 μl.

### Determinations of fatty acids

Fatty acids were extracted according to the Folch method (Folch et al. 1957). Chromatographic separation of methyl esters was carried out on a gas chromatograph (Hewlett Packard 5890, USA) with a flame ionization detector at 260°C, using “split/splitless” injection (1:50). A capillary column (Innowax 30 m × 0.25 mm × 0.25 μm) was used as a stationary phase and helium gas as a carrier gas (1.5 ml min^−1^). Chromatographic separation was achieved in a temperature ramp from 60 to 200°C at a rate of 12°C min^−1^, and the final temperature was maintained for 20 min. The injector temperature was 240°C. Identification of acids was based on retention times of internal standards (Supelco 37 Component Fame Mix, USA). However, quantitative interpretation was based on peak areas as compared to an internal standard–heptadecanoic acid methyl ester*.*

### Kinetics of denitrification

During the initial experiments several calculations were carried out in order to determine the most suitable model. Based on the obtained data, it was concluded, that the zero order kinetic equation was most fit for further modeling (Foglar et al. 2005; Dhamole et al. 2007).

## Results and discussion

### The effect of the addition of compatible solutes on the activity of enzymes in denitrification and fat degradation

The consortium used to carry out the denitrification process was identified by restriction fragment length polymorphism of 16S rRNA gene amplicons and sequencing. It was found to consist of 12 dominant bacterial strains with the closest match to strains such as: *Alacligenes faecalis, Pseudomonas alcaligenes, Pseudomonas putida, Klebsiella oxytoca, Stenotrophomonas maltophilia, Defluvibacter lusatiae, Hafnia alvei, Microbacterium foliorum, Microbacterium phyllosphaerae, Microbacterium profundi, Ochrobactrum intermedium, Sphingobacterium* sp. All the isolated bacteria were capable of nitrate reduction. *Alcaligenes faecalis, Pseudomonas alcaligenes, Pseudomonas putida, Klebsiella oxytoca, Stenotrophomonas maltophilia, Ochrobactrum intermedium, Sphingobacterium* sp. are well known for the ability to completely reduce nitrate to elemental nitrogen. None of the described microorganisms could synthesize ectoine, however some *Pseudomonas putida* species were previously reported to be capable of betaine production ((Kets et al. 1996). Trehalose could be synthesized by *Stenotrophomonas maltophilia* (Roder et al. 2005).

The wastewater denitrification process was conducted after the addition of 1 mM compatible solutes (trehalose or ectoine) and a fatty substance (1.5 g l^−1^) isolated from oiled bleaching earth, and their effect on the rate of denitrification was determined. On the first day denitrification, the bacteria adapted to a new environment (lag phase) and no decrease in the nitrate level was observed. The nitrite concentration, which is an intermediate denitrification product, increased slightly in all cultures at that time, but did not exceed 8 mg N l^−1^. A considerable increase in the nitrate reduction rate was observed on the subsequent days. In particular, the addition of ectoine accelerated the process, and caused the complete removal of nitrates after 5 d of the process, and that of nitrites after 7 d. The addition of trehalose did not affect significantly the acceleration of the denitrification process; it proceeded as in wastewater without the addition of any compatible solutes. Although the nitrates were removed completely on the 7th day (on the 8th day in wastewater without compatible solutes), the complete removal of nitrite occurred in both cases on the 9th day of the process. However it is also plausible that the lag phase could be explained otherwise. The shape of the nitrate degradation curve could be the result of the microbial consortium acclimation to either salinity or nitrous acid. Additionally the base producing nature of denitrification processes increased the pH, making the solution more basic over the course of reaction. This increase in pH may have created an environment more conducive to denitrification, increasing the rate of the reaction after an initial lag. It has been shown by other researchers that denitrification occurs more rapidly at elevated pH values (Rust et al. 2000). In this conditions nitrite peaking may have presented some nitrous acid toxicity, although this seems unlikely. The pH was monitored during all experiments when cultures were terminated, so the phosphate buffer was strong enough to keep the pH in range 7.2–8.1 and from spectacular rising pH during denitrification.

**Fig. 1:**
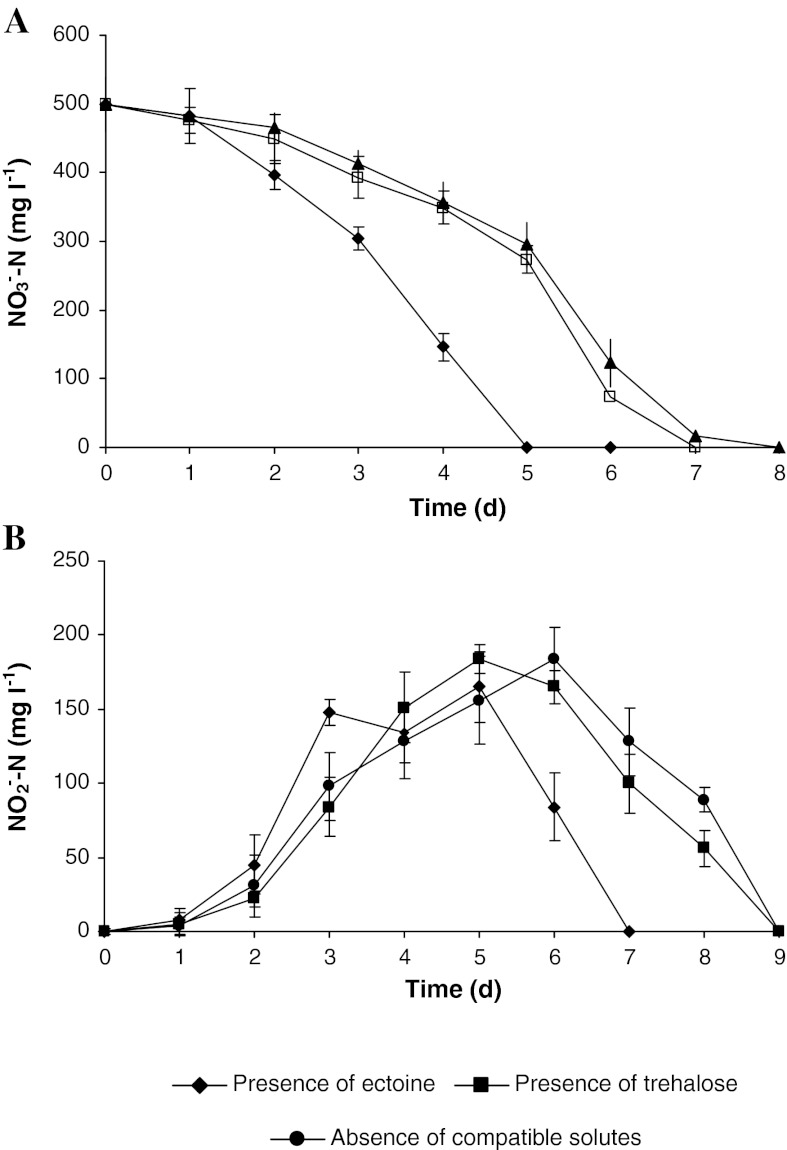
Effect of compatible solutes (ectoine and trehalose) on **a** nitrate and **b** nitrite concentration in wastewater

In all cultures, the maximal nitrite concentration did not exceed 185 mg N l^−1^ (Fig. 1). These results confirm the calculated specific nitrate reduction rate, which was: 5.7 mg N g^−1^ VSS h^−1^ for denitrification in the presence of ectoine, 3.4 mg N g^−1^ VSS h^−1^ with trehalose, and 3.2 mg N g^−1^ VSS h^−1^ without any compatible solutes addition (Table 2). During the denitrification with a hydrophilic carbon source (methanol, acetic acid) in a sodium chlorate-free environment, considerably higher values of specific nitrate reduction rate, above 100 mg N g^−1^ VSS h^−1^ were obtained (Foglar et al. 2005; Rodríguez et al. 2007). The salinity of wastewater decreased the value of the specific nitrate reduction rate to 12–23 mg N g^−1^ VSS h^−1^ (Glass and Silverstein 1999; Peyton et al. 2001).

**Table 2:** Specific nitrate reduction rate, removal efficiency of nitrate–N and zero order rate constant for nitrate reduction for the denitrification process of wastewater

Parameter		Absence of compatible solutes	Ectoine	Trehalose
End concentration of DM in wastewater	g l^−1^	0.80 ± 0.12	0.76 ± 0.11	0.84 ± 0.14
Maximum concentration of nitrite	mg N l^−1^	182 ± 12	162 ± 21	181 ± 14
Efficiency	%	100	100	100
Specific nitrate reduction rate	mg N g^−1^ VSS h^−1^	3.25 ± 0.13	5.73 ± 0.23	3.45 ± 0.15
Zero order rate constant for nitrate reduction	mg N l^−1^ h^−1^	72 ± 7	94 ± 6	70 ± 8

Initial concentration of DM in wastewater = 0.05 ± 0,01 g l^−1^

When fatty substances are being used as a carbon source, the key factors influencing denitrification are the activities of enzymes participating in nitrate reduction (nitrate reductase) and fat hydrolyzing enzymes (lipases). Nitrate reductase is an enzyme located in the bacterial cell membrane and determines the first stage of denitrification, that is reduction of nitrates to nitrites (Kim et al. 2006). Lipases are enzymes secreted outside the cell and are responsible for fat hydrolysis to glycerol and fatty acids. These enzymes have great catalytic potential, and require an insoluble substrate at the fat–water phase boundary (Gupta et al. 2004). They determine the supply of the organic carbon source (electron donor) essential for denitrification. It is of particular importance if high molecular weight compounds of hydrophobic character are used as a carbon source.

**Fig. 2:**
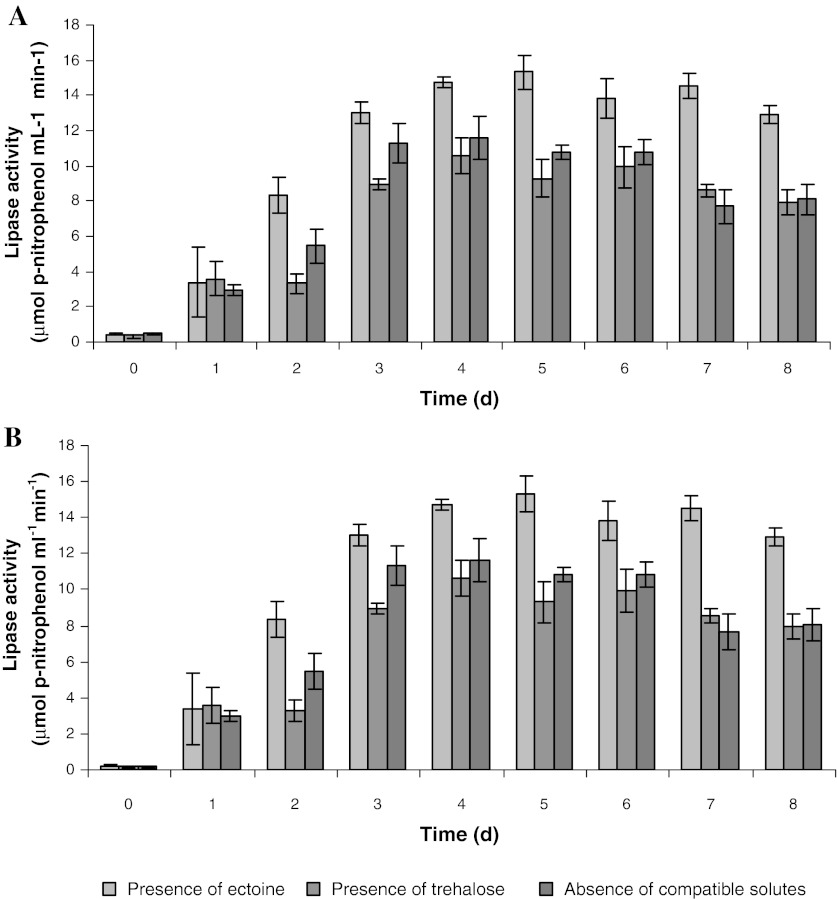
Effect of compatible solutes (ectoine and proline) on lipase (**a**) and nitrate reductase (**b**) activities during denitrification process

The measured activities of nitrate reductase and lipase confirm the important effect of ectoine on the denitrification process. At the beginning (first day), the activity of lipases did not exceed 3 μM p-nitrophenol ml^−1 ^min^−1^. Subsequently, the activity increased and already on the second day reached 9 μM p-nitrophenol ml^−1 ^min^−1^. The highest lipase activity was obtained on the fifth day (15.8 μM p-nitrophenol ml^−1 ^min^−1^). This activity remained until the end of the denitrification process. Lipase activity was similar during denitrification with the addition of trehalose, and denitrification without compatible solutes. The enzyme activity also increased from the initial 3 μM p-nitrophenol ml^−1 ^min^−1^ (first day) until the fourth day (lipase activity: 11–12 μM p-nitrophenol ml^−1 ^min^−1^). However, these activities were lower by 32%, than during denitrification with ectoine (Fig. 2a). Changes in nitrate reductase were similar. Initially no activity of this enzyme was observed. It resulted from the fact that the bacteria used for denitrification were grown under aerobic conditions, and needed time for adaptation to new environmental conditions, including synthesis of cytochrome aa_3_, which transfers electrons onto nitrate in the respiratory chain. The activity of nitrate reductase during denitrification with ectoine addition increased until the fourth day of the process, reaching a maximum of 49 mg N ml^−1 ^min^−1^. The increase in nitrate reductase activity in cultures with trehalose and without the addition of compatible solutes proceeded similarly to denitrification with ectoine addition, but the activity was lower by 35% (Fig. 2b). A similar phenomenon was observed by Werber and Mevarech (1978), but they measured the activity of reductases in the genus isolated in the Dead Sea. The authors stated that after transferring the *Halobacteria* from aerobic to anaerobic conditions, nitrate reductase gene expression occurred at 85 h of culturing, and its activity was 30 mg N min^−1^ g^−1^ protein. The enzyme activity increased rapidly, and at 200 h of culturing reached 400 mg N min^−1^ g^−1^ protein.

**Fig. 3:**
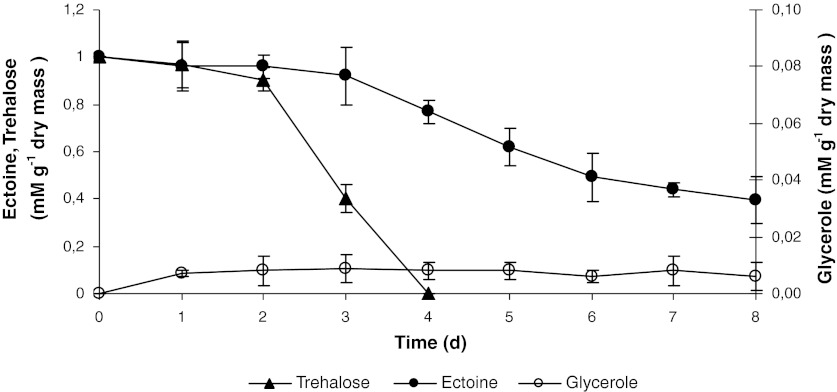
Changes of ectoine, trehalose and glycerole concentrations during the denitrification process

Changes in ectoine and trehalose concentration in wastewater were observed during the denitrification process (Fig. 3). Until the second day of the process, the concentration of both compounds in wastewater did not differ significantly and was 1 mM. After the second day, the concentration of trehalose began to decrease considerably, and on the fourth day the compound was no longer observed in wastewater. The ectoine concentration decreased on the 8th day to 0.1 mM. The level of ectoine in cell biomass was 0.16 mg per 1 g of dry mass, which is 80% of ectoine contained in wastewater at the end of the process. As the final ectoine concentration in wastewater was 0.1 mM, the amount of metabolized ectoine, used as carbon source was 10%. No trehalose was found in the bacterial biomass; thus, it must be acknowledged that this compound was fully used as a carbon source during the denitrification process. The fact that glycerol was produced during fat hydrolysis, and that glycerol is a substance included among compatible solutes, and at the same time a perfect carbon source for denitrification, is worth mentioning. Its small concentration in wastewater, below 0.003 mM, and in the cell biomass (below 0.06 mM) is evidence that this compound was used immediately by the bacteria for energetic processes and metabolized fully.

The results on the trehalose and ectoine content in the cell biomass indicate that denitrifying bacteria preferred amino acids (ectoine) as a compatible solute, while they used sugars (trehalose) only as a carbon source and metabolized them. It should be emphasized that the ability to accumulate and metabolize compatible solutes applies to numerous heterotrophic bacteria, cyanobacteria, phototrophic bacteria, methane bacteria, fungi and yeast (Welsh 2000), including bacteria that do not possess the ability to produce them (Jebbar et al. 1992; Nagata and Wang 2005). Studies of Zhang et al. (2008) can serve as an example. They used ectoine to increase the efficiency of ethanol fermentation conducted by the bacterium *Zymomonas mobilis*. It was observed that relative enzyme activities of glucokinase (GK), glucose-6-phosphate dehydrogenase (G-6-PDH), and alcohol dehydrogenase (ADH) increased by 29.9, 11.6, and 7.7%, respectively, in the presence of 0.5 mM ectoine. This resulted in an increase in volumetric ethanol productivity in the presence of 1 mM ectoine to 1.1 g l^−1 ^h^−1^, which was higher than in the absence of ectoine (0.7 g l^−1 ^h^−1^). Examples of the use of ectoine as a protective substance in enzymatic processes are known. According to Wang and Zhang (2010) to improve the production of biodiesel by enzymatic conversion of triglycerides in cottonseed oil, compatible solutes were added to the solvent-free methanolysis system to prevent competitive methanol inhibition of the immobilized lipase (Novozym^®^ 435). The results indicated that the addition of ectoine increased biodiesel synthesis using a three-step methanol addition process. The concentration of methyl ester (ME) reached a maximum of 95% in the presence of 1.1 mM ectoine, which constituted an increase by 20.9% compared to that in the absence of ectoine. Bacteria used for denitrification did not have the ability to produce ectoine and trehalose, and the amounts that they accumulated from the environment were much smaller than in halotolerant or halophilic bacteria, which are capable of growing in a high salinity environment (from 3 to 18% NaCl) (Onraedt et al. 2004). Such high osmotic potentials force microorganisms to accumulate compatible solutes in the amounts directly proportional to the salinity of the environment.

### Fatty acid biodegradation

**Fig. 4:**
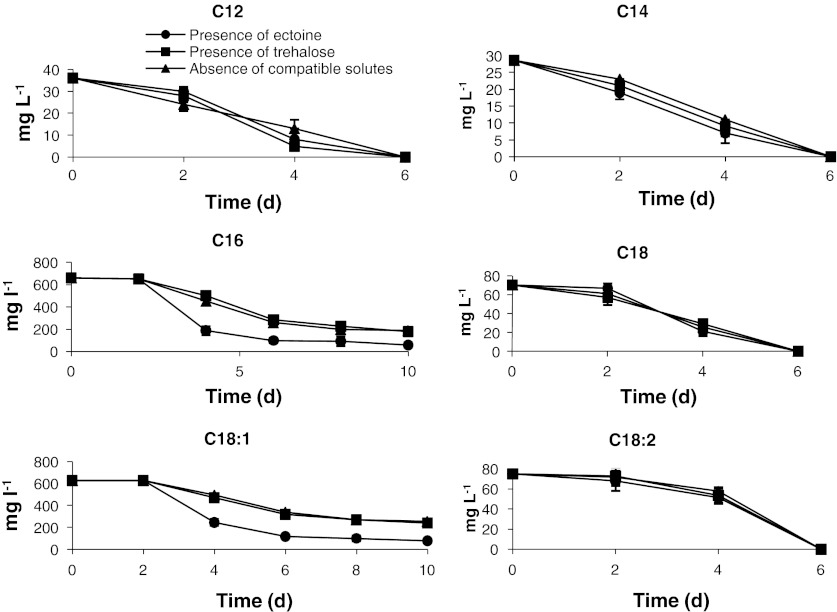
Changes of fatty acids concentrations during the denitrification process

Fatty acids with chains containing more than four carbon atoms have a limited solubility in water, and those with more than twelve carbon atoms are practically insoluble. Fatty acids form a monomolecular layer on the surface of water, with the carboxyl groups directed towards water (Ron and Rosenberg 2002). Fat degradation products, such as glycerol or fatty acids are, for many types of microorganisms, a perfect source of carbon and energy. Heterotrophic denitrifying bacteria need an organic carbon source to conduct life processes (growth, respiration). Carbon sources used in waste treatment technology are very diverse; for instance, methanol, ethanol, glucose, acetate, formic acid, and industrial wastes, including molasses, whey, wastewater, and even cellulose (Mateju et al. 1992). The use of a hydrophobic carbon source enabled the total removal of nitrate removal from wastewater. Fatty acids with the shortest chains were metabolized first during denitrification (Fig. 4). C12 and C14 fatty acids, at initial amounts of 36 and 29 mg l^−1^, respectively, were removed completely in all cultures already on the sixth day of the process. Bacteria also used acids of higher carbon atom numbers (C18 and C18:2). The initial concentration of these acids in wastewater was 70 and 75 mg l^−1^, respectively. On the sixth day of the process, a complete removal of these fatty acids from wastewater was observed. The fatty acids that prevailed in wastewater (C16 and C18:1) were biodegraded faster when ectoine was added. On the sixth day of the process, in the culture medium containing ectoine, the C16 acid concentration decreased from the initial 660 to 175 mg l^−1^, while the C18:1 acid concentration dropped from 625 to 245 mg l^−1^.

## Conclusions

The presented results confirm a significant effect of ectoine on the denitrification rate. Ectoine increased lipase activity, thus the availability of organic compounds for the process increased, and influenced denitrification by increasing the nitrate reductase activity. No significant effect of trehalose on the denitrification process was observed in the study. It was confirmed that fatty acids constitute a perfect carbon source for the denitrification process. Therefore, it is possible to conduct an effective denitrification along with the simultaneous biodegradation of fatty acids isolated from oiled bleaching earth in an environment of high osmotic potential.
